# Stochastic factors drive dynamics of ammonia-oxidizing archaeal and bacterial communities in aquaculture pond sediment

**DOI:** 10.3389/fmicb.2022.950677

**Published:** 2022-10-06

**Authors:** Lili Dai, Liqin Yu, Liang Peng, Ling Tao, Yanbin Liu, Gu Li

**Affiliations:** ^1^Key Laboratory of Freshwater Biodiversity Conservation, Ministry of Agriculture and Rural Affairs, Yangtze River Fisheries Research Institute, Chinese Academy of Fishery Sciences, Wuhan, China; ^2^College of Fisheries, Huazhong Agricultural University, Wuhan, China; ^3^Ningxia Fisheries Research Institute Co., Ltd., Yinchuan, China

**Keywords:** *amoA*, community dynamics, stochastic processes, disturbance, areas

## Abstract

Ammonia-oxidizing archaea (AOA) and bacteria (AOB) play an important role in nitrification, which is essential in the global nitrogen cycle. However, their dynamics and the underlying community processes in agricultural ecosystems under disturbance remain largely unknown. In this study we examined the spatiotemporal dynamics of AOA and AOB communities and analyzed their community processes in the sediment of aquaculture ponds across three different areas in China. We found some significant temporal changes in AOA and AOB community diversity and abundances, but no temporal changes in community composition, despite the significant variations in sediment properties between different sampling times. Nevertheless, significant differences were found for AOA and AOB communities between different areas. Distinct area-specific taxa were detected, and they were found to be important in determining the response of AOA and AOB communities to environmental factors. In addition, geographic distance was found to be significantly correlated with AOA and AOB community composition, which demonstrates that dispersal limitation could significantly contribute to the variations in AOA and AOB communities, and stochastic processes were found to be important in structuring AOA/AOB communities in aquaculture ponds. Taken together, our study indicates that the dynamics of AOA and AOB are based on their community characteristics in aquaculture pond sediment. Our results, for the first time, provide evidence for the dynamics of AOA and AOB communities being driven by stochastic factors in a disturbed environment, and might also be of use in the management of the aquaculture environment.

## Introduction

The process of nitrification plays a central role in the global nitrogen cycle. This process consists of two important steps whereby ammonia is oxidized to nitrate *via* nitrite. Ammonia-oxidizing archaea (AOA) and bacteria (AOB) perform the process of ammonia oxidation which constitutes the first step of nitrification and is the rate-limiting step. Both groups use ammonia as their sole energy source (Konneke et al., 2014; Lehtovirta-Morley et al., 2016), although some of them are reported to be able to assimilate amino acids (Mussmann et al., 2011). As a result of their importance in nitrogen cycling and their potential use in ammonia removal, for instance through coupled nitrification–denitrification processes, AOA and AOB have been well-studied in various habitats, including oceans (Francis et al., 2005; Sintes et al., 2015), estuaries (Zheng et al., 2014; Smith et al., 2015), lakes (Liu and Yang, 2021), soils (Pester et al., 2012; Shen et al., 2021), and wetlands (Wang et al., 2013, 2019). For example, many factors have been shown to affect the composition and abundance of AOB and AOA, including pH, temperature, salinity (Santos et al., 2020), ammonia concentration (Taylor et al., 2012), nutrient levels (Dai et al., 2018), competition (French et al., 2021), and predators (Kim et al., 2019).

Despite the enormous amount of research on the distribution and activity of ammonia oxidizers, these studies have mainly either focused on a particular regional scale or been for specific seasons. However, these microorganisms are subjected to both spatial and temporal environmental factors in agricultural environments. Indeed, there are still many uncertainties concerning AOA and AOB communities in these environments, as they are not necessarily correlated to specific environmental factors (Wang et al., 2019). Factors that control nitrifying communities are found to vary spatiotemporally (Bernhard et al., 2019). Understanding how ammonia-oxidizing communities in agricultural environments, especially under major disturbance, would be affected by changes of spatial and temporal factors would thus be important for understanding their behavior, and might also help improve, for example, the selection of optimal microbiomes for microbial communities that perform better in a desired process (Wright et al., 2019).

On the other hand, both deterministic and stochastic processes have been used to explain variations in microbial community composition (Vellend and Agrawal, 2010; Barnett et al., 2020). In deterministic processes, abiotic and/or biotic factors determine the community assembly, while in stochastic processes, probabilistic dispersal and random dynamics are observed (Vellend and Agrawal, 2010; Chase and Myers, 2011). Deterministic processes have historically been well-studied to explain the variation in community composition at different scales (Taylor et al., 2012; Jiao and Lu, 2020). The importance of stochasticity, especially in highly disturbed environments, has recently also been proved (Chase and Myers, 2011; Barnett et al., 2020). But how these processes combine to influence the dynamics of ammonia oxidizers has rarely been studied.

Aquaculture ponds, which contributed to 52% of the freshwater aquatic products in China in 2020 (Bureau of Fisheries et al., 2021), are unique aquatic ecosystems characterized by profound disturbance in the sediment as a result of fish activities and nutrient accumulation during the rearing of aquatic organisms. Indeed, the sediment could become highly reduced during the culture periods with the decomposition of organic matter, and cause significant effects on the nitrification process (Dai et al., 2018). Although effects of environmental characteristics on microbial community variation have been found during disturbance (Guggenheim et al., 2020; Monteiro et al., 2020), different responses of microbial communities to environmental change due to adaption has also been detected (Dai et al., 2018; Xia et al., 2020; Zhang et al., 2021). In addition, research on agricultural cropland has indicated a significant influence of stochastic processes besides deterministic processes on bacterial community assembly (Barnett et al., 2020). A recent study on large-scale disturbances on nitrifying microbes also found region-specific patterns of AOA and AOB, and indicated significant role of regional drivers (Bernhard et al., 2019). These findings suggest that a combination of stochastic and deterministic processes might drive microbial community dynamics in environment under disturbance. However, the processes that drive microbial community dynamics in the aquaculture environment remain largely unknown.

The ammonia monooxygenase subunit A (*amoA*) gene has been widely used to study ammonia oxidizers, and is considered to be second only to the 16S rRNA gene as the most frequently sequenced maker gene in microbial ecology studies (Alves et al., 2018). In addition, it has been acknowledged that a finer resolution of phylogeny can be provided by using the *amoA* gene (Wang et al., 2021). In this study, to reveal the spatiotemporal dynamics of AOA and AOB communities in response to environmental changes in aquaculture pond sediment, we analyzed *amoA* gene dynamics in the sediment of aquaculture ponds across three different representative aquaculture areas in China. These ponds were stocked with similar fish species, and were under similar common fish-rearing management to exclude the effects of fish activities on ammonia oxidizers. We hypothesized that the dynamics of ammonia-oxidizing communities are induced by both stochastic and deterministic processes in aquaculture pond sediment. The main objectives of our study were (i) to examine the spatiotemporal dynamics of AOA and AOB communities in aquaculture pond sediment, (ii) to reveal the potential drivers of the dynamics of AOA and AOB communities in aquaculture pond sediment, and (iii) to study the community assembly processes underlying the community dynamics of ammonia oxidizers in aquaculture ponds. The results of our study would further the understanding of the actual dynamics of ammonia-oxidizing communities, and might also be of use in the management of the aquaculture environment.

## Materials and methods

### Study sites and sample collection

Aquaculture ponds used to rear grass carp (*Ctenopharyngodon idellus*) in three areas of China—Changjiang (CJ), Heilongjiang (HLJ), and Zhujiang (ZJ) were selected for study. These ponds were all earth-based and under similar fish-rearing management. The distances between the areas are about 2,200, 870, and 2,890 km between CJ and HLJ, CJ and ZJ, and HLJ and ZJ, respectively. General information about these ponds is provided in Table 1. Surface sediment samples from three different ponds in each area were collected with a core sampler in May (Sampling Time T1, beginning of fish culture period), August (Sampling Time T2, middle of culture period), and October (Sampling Time T3, end of culture period) in 2017, respectively. In each pond, three to five sediment samples from different locations distributed evenly around the pond center were mixed to form one composite sample, which was immediately transferred to the laboratory. At the laboratory, the composite samples were either air-dried for physicochemical analysis or kept below −70°C until DNA extraction.

**TABLE 1:** General information about the sampling ponds.

Sampling sites	Location	Fish breeds	Total sampling area (hm^2^)	Stocking density (kg/hm^2^)	Water depth (m)
Changjiang ponds	Jingzhou, Hubei (30°18′N, 112°03′E)	*C. idellus* inter-cropped with *H. nobilis* and *H. molitrix*	8	∼7,500	∼1.5 to ∼3.5
Heilongjiang ponds	Suihua, Heilongjiang (46°23′N, 126°44′E)		26	∼9,750	
Zhujiang ponds	Zhongshan, Guangdong (22°36′N, 113°30′E)		45	∼6,000	

### Physicochemical analysis

Water temperature (WT) was measured *in situ* with a handheld Multiparameter (*In Situ* Inc., United States). Sediment pH was measured with a pH meter using a 1:2.5 (m/v) ratio of sediment and water. Total nitrogen (TN) and total phosphorus (TP) concentrations were determined by the semi-micro Kjeldahl method and the alkali fusion Mo-Sb anti-spectrophotometry method, respectively. Nitrate (NO_3_^–^), nitrite (NO_2_^–^), and ammonium (NH_4_^+^) contents were measured by extracting with 1 mol/L KCl solution and quantifying with a spectrophotometer. Total organic carbon (TOC) content was determined by the sulfuric acid and potassium dichromate oxidation method. Microbial biomass carbon (MBC) content was determined by the fumigation-extraction method to reflect the total sediment microorganisms, and the dissolved organic carbon (DOC) content was determined from the non-fumigated fraction. Contents of β-glucosidase (Gal), urease (Ure), acid-phosphatase (Pho), and arylsulfatase (Ary) were also determined by incubating with different substrates and calculating the substrate variation to evaluate the level of organic matter transformation. Potential ammonia oxidation rate (PNR) in sediment was determined using the chlorate inhibition method to reflect the potential ammonia oxidizing activity of ammonia oxidizers.

### DNA extraction and *amoA* gene sequencing

DNA was extracted from approximately 1 g sediment sample with the DNeasy PowerSoil Kit (QIAGEN, Hilden, Germany), according to the manufacturer’s instructions. The *amoA* genes of AOA and AOB were amplified by polymerase chain reaction (PCR) with primers Arch-amoAF (5′-STAATGGTCTGGCTTAGACG-3′)/Arch-amoAR (5′-GCGGCCATCCATCTGTATGT-3′) (Francis et al., 2005) and amoA-1F (5′-GGGGTTTCTACTGGTGGT-3′)/amoA-2R (CCCCTCKGSAAAGCCTTCTTC) (Rotthauwe et al., 1997), respectively. The reaction conditions for AOA were: 95°C for 5 min; 32 cycles of 95°C for 45 s, 58°C for 45 s, 72°C for 1 min; and 72°C for 10 min. The reaction conditions for AOB were: 95°C for 30 s; 35 cycles of 95°C for 5 s, 58°C for 40 s, 72°C for 70 s; and 80°C for 20 s. The amplified products were then used for clone library construction. More than 50 colonies for each clone library were sequenced on the ABI 3730xl platform (Applied Biosystems, Foster City, CA, United States).

### Quantification of *amoA* genes

The abundances of *amoA* gene copies were determined by quantitative fluorescence PCR (qPCR) using the same primers as above. Each qPCR was performed in duplicate in 20 μL reaction solution, which contained 10 μL SybrGreen qPCR Master Mix, 10–40 ng template DNA, and 0.4 μL of 10 μM forward and reverse primers. The reaction conditions for AOA quantification were 95°C for 5 min, followed by 45 cycles of 95°C for 30 s, 53°C for 38 s, and 72°C for 45 s; and the conditions for AOB were 95°C for 3 min, followed by 45 cycles of 95°C for 15 s, 57°C for 20 s, 72°C for 30 s. Plasmids from AOA and AOB clones were used as standards for quantification of *amoA* genes of AOA and AOB, respectively.

### Bioinformatics and statistical analysis

The *amoA* gene sequences from each sample were combined and screened for short or artifact sequences, which were then discarded. The remaining sequences were then blasted in GenBank using the BLAST tool,^1^ and sequences with low similarity to existing *amoA* gene sequences were discarded. The remaining high-quality sequences were clustered into operational taxonomic units (OTUs) based on a similarity threshold of 97% (Pester et al., 2012; Shen et al., 2021). Calculation of alpha diversity, including *chao1*, *Shannon*, and *simpson* indices, as well as the coverage and rarefaction analyses for each clone library were conducted using mothur v.1.39.5 software (Schloss et al., 2009). Operational taxonomic units were aligned with known taxa sequences in the CLUSTALX program (version 2.0.11), and then used in MEGA software (version 7.0.26) to construct a neighbor-joining tree using bootstrap support levels of 1,000 resampled datasets. The sequencing coverage was found to be more than 95% for both AOA and AOB in all the samples, and few extra OTUs were detected when we tried to sequence more colonies for each clone library. Rarefaction analyses also indicate sufficient sequencing depth for both AOA and AOB colonies.

Principal coordinates analysis (PCoA) was performed using the ape package (Paradis and Schliep, 2019) to analyze community profiles based on the weighted UniFrac distance. Analysis of similarity (ANOSIM) was performed using the vegan package (Oksanen et al., 2020) to determine the statistical differences between different groups based on the Bray-Curtis distance. Linear discriminant analysis (LDA) was performed through an online toolkit^2^ to identify distinct OTUs among different groups with alpha values less than 0.05 for a factorial Kruskal-Wallis test and LDA scores > 2.0. Co-occurrence networks were determined to reveal interactions between OTUs and possible keystone taxa were identified by Spearman’s correlation analysis in the psych package (Revelle, 2022), and these networks were then visualized in Gephi (version 0.9.1). Distance-based redundancy analysis (db-RDA) and canonical correlation analysis (CCA) were performed using the vegan package to detect relationships between AOA and AOB communities and sediment properties. Distance-based Moran’s eigenvector maps (dbMEM) created with the adespatial package (Dray et al., 2022) were used to generate spatial factors (MEM_*i*_) in order to determine the effects of spatial distance on ammonia oxidizers based on the Cartesian coordinates of each site calculated from the latitude and longitude values.

Correlation heatmaps between OTUs and sediment properties were constructed with the psych package based on the Pearson correlation. The β-nearest taxon index (β-NTI) was calculated using the picante package (Kembel et al., 2010) to evaluate the phylogenetic community assembly from the deviation of the observed β-mean nearest taxon distance (β-MNTD) from the null β-MNTD, which was created with 999 community randomizations. Values of β-NTI between −2 and 2 indicate a stochastic process on community assembly, and values > 2 or < −2 indicate a deterministic process. All these analyses were performed within the R environment (version 4.1.3). Plots were performed using the Graphpad prism software (version 7.00) or the ggplot2 package v. 3.3.5 (Wickham, 2016).

Statistical differences between physicochemical characteristics on the one hand and *amoA* gene copy numbers and diversity on the other were analyzed using the IBM SPSS software (version 19.0) based on samples from the same sampling times/areas in order to avoid any masking effect. All data are expressed as mean ± standard error (SEM). Data homogeneity was tested with Levene’s test, and the normality of variance was tested with the Kolmogorov–Smirnov test. If the Kolmogorov–Smirnov test failed, the data were subjected to logarithmic transformation. The data were then subjected to one-way analysis of variance (ANOVA) followed by Tukey’s test. Values of *p* < 0.05 indicate significant differences between different samples.

## Results

### Physicochemical characteristics in aquaculture pond sediments

Water temperature varied significantly between different sampling times in all the sampling areas (*p* < 0.001). In both CJ and HLJ pond sediments, significant differences in NO_2_^–^, NO_3_^–^, and MBC content were found between different sampling times, and TN and TOC concentrations in all the sampling areas varied significantly between different sampling times (Supplementary Figure 1). Generally, TN concentration decreased during the middle of the fish culture period, and TOC concentrations were elevated during the fish culture. Concentrations of TN, TOC, and β-glucosidase content varied significantly between different sampling areas, and concentrations of urease and arylsulfatase were also significantly different between sampling areas, with their concentrations being the highest in ZJ ponds and the lowest in CJ ponds (Supplementary Figure 2).

### Ammonia-oxidizing archaea and ammonia-oxidizing bacteria community structure variations in aquaculture pond sediments

**FIGURE 1:**
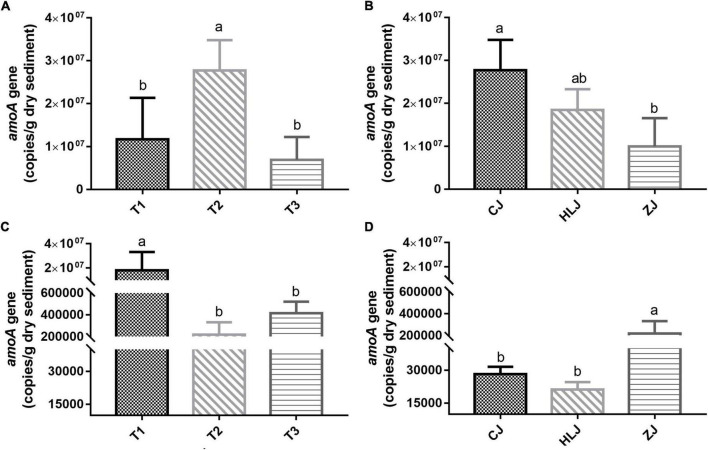
Numbers of ammonia-oxidizing archaea (AOA) *amoA* gene copies in Changjiang (CJ) ponds **(A)** and ammonia-oxidizing bacteria (AOB) in Zhujiang (ZJ) ponds **(C)** at different sampling times, and AOA **(B)** and AOB **(D)** copies in different sampling areas at sampling time T2. The error bar denotes standard error among the samples, and different letters above bars indicate significant difference (*p* < 0.05, *n* = 3). Only ponds with significant differences are presented.

The abundances of *amoA* gene copies of AOA in CJ pond sediments varied significantly between T2 and other sampling times, and the abundances of *amoA* gene copies of AOB in ZJ pond sediments varied significantly between T1 and other sampling times (Figures 1A,C). However, no significant differences in AOA or AOB *amoA* gene copy numbers were found between sampling times in other ponds. On the other hand, although there was no significant difference in *amoA* gene copy numbers between different areas at the beginning of the fish culture period, abundances of *amoA* gene copies of AOA and AOB varied significantly among different sampling areas at sampling time T2 (Figures 1B,D).

A significant difference in *chao1* was detected in CJ AOA samples between T2 and other sampling times, and the *shannon* index was also detected to vary significantly in HLJ AOA samples between T2 and other sampling times (Table 2). The *shannon* and *simpson* indexes of AOB in HLJ ponds varied significantly between T1 and other sampling times. However, few other significant differences were found between different sampling times. Significant differences in AOA diversity between different sampling areas were found at all the sampling times. In contrast, no significant difference in AOB diversity between different sampling areas was found at sampling time T1, but differences in *chao1*, *Shannon*, and *simpson* of AOB were significant at other sampling times.

**TABLE 2:** Microbial diversity of different pond sediment samples at different sampling times*.

Sampling time	AOA			AOB		
	*chao1*	*shannon*	*simpson*	*chao1*	*shannon*	*simpson*
**Changjiang**						
May	10.6 ± 1.2^b^	1.6 ± 0.2	0.3 ± 0.1^a^	4.7 ± 2.1^a^	0.8 ± 0.7	0.6 ± 0.3
August	24.9 ± 5.4^a^	1.6 ± 0.4	0.3 ± 0.1^ab^	6.0 ± 5.2^ab^	0.7 ± 0.7	0.7 ± 0.3
October	10.5 ± 1.3^b^	1.0 ± 0.3	0.5 ± 0.2^b^	15.5 ± 6.1^b^	1.7 ± 0.3	0.3 ± 0.1
**Heilongjiang**						
May	7.2 ± 2.3	1.0 ± 0.4^b^	0.5 ± 0.2	12.7 ± 6.7	1.4 ± 0.6^a^	0.3 ± 0.2^a^
August	15.0 ± 6.5	1.7 ± 0.2^a^	0.3 ± 0.1	22.6 ± 10.4	2.4 ± 0.2^b^	0.1 ± 0.0^b^
October	10.0 ± 7.0	0.9 ± 0.3^b^	0.5 ± 0.1	19.1 ± 4.8	2.4 ± 0.1^b^	0.1 ± 0.0^b^
**Zhujiang**						
May	20.5 ± 1.5	2.0 ± 0.3	0.2 ± 0.1	15.1 ± 4.2^a^	1.5 ± 0.3^a^	0.3 ± 0.1
August	18.5 ± 4.4	2.4 ± 0.2	0.1 ± 0.0	5.5 ± 2.8^b^	1.1 ± 0.2^b^	0.4 ± 0.1
October	13.7 ± 6.8	2.0 ± 0.4	0.2 ± 0.1	9.2 ± 3.0^ab^	1.3 ± 0.1^ab^	0.4 ± 0.0

*Different superscript letters indicate significant differences between samples at different times (*p* < 0.05, *n* = 3).

**FIGURE 2:**
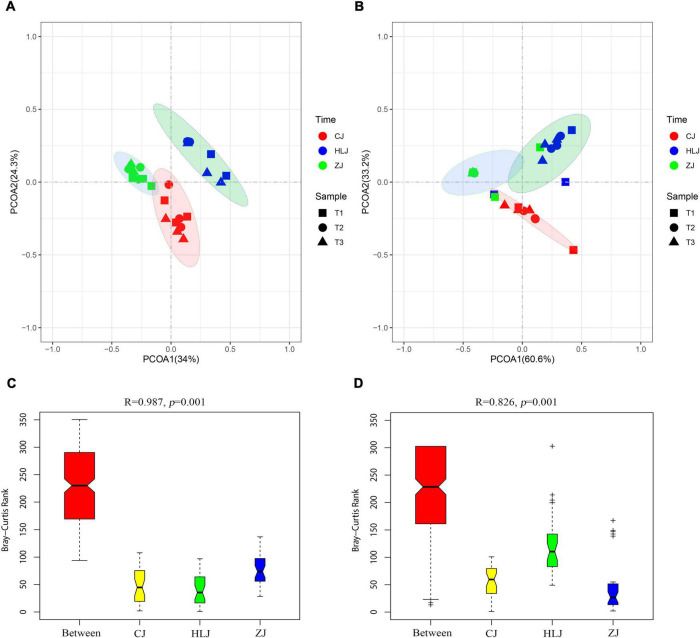
Principal coordinates analysis (PCoA) plots of the ammonia-oxidizing archaea (AOA) **(A)** and ammonia-oxidizing bacteria (AOB) **(B)** community using the weighted UniFrac distance metric, and analysis of similarity (ANOSIM) analysis based on Bray-Curtis distances of AOA **(C)** and AOB **(D)** community dissimilarity/similarity from sediment samples in different areas. Ellipses were drawn for AOA and AOB with confidence intervals of 95 and 75%, respectively. T1–3 represent different sampling times, and CJ, HLJ, and ZJ represent sediment samples collected in different areas.

Neither AOA nor AOB communities from different sampling times could be separated according to the PCoA; however, AOA and AOB communities in different sampling areas could be clearly separated at confidence levels of 95 and 75%, respectively (Figures 2A,B). Analysis of similarity (ANOSIM) based on Bray-Curtis distances indicated no significant difference between AOA or AOB communities from different sampling times in CJ, HLJ, or ZJ ponds, but there were significant differences in AOA (Figure 2C) and AOB (Figure 2D) communities between different sampling areas.

### Correlations between ammonia-oxidizing archaea and ammonia-oxidizing bacteria communities in aquaculture pond sediments

**FIGURE 3:**
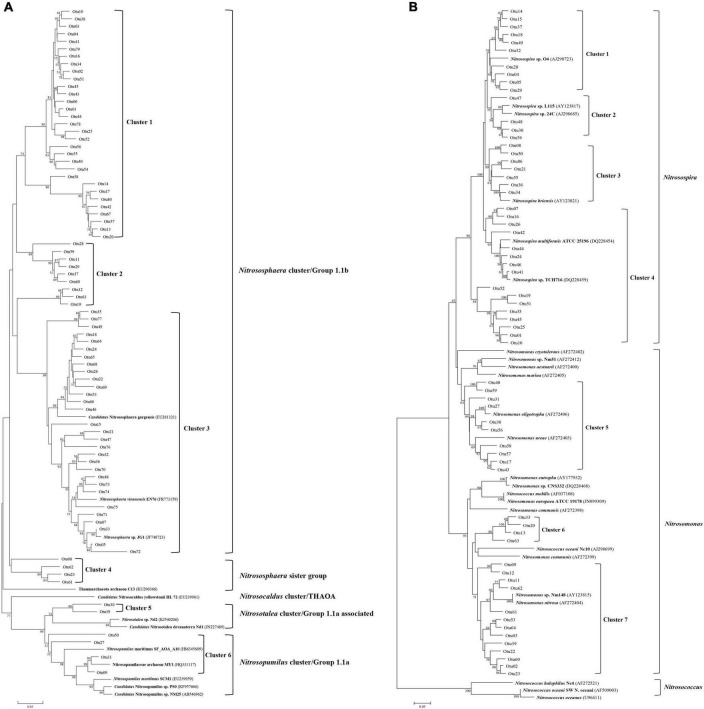
Phylogenetic clusters of ammonia-oxidizing archaea (AOA) **(A)** and ammonia-oxidizing bacteria (AOB) **(B)** operational taxonomic units (OTUs) based on neighbor-joining analysis with known AOA and AOB taxa from GenBank. Numbers at the nodes indicate bootstrap support levels of 1,000 resampled datasets, with only those bootstrap values larger than 50% being presented.

**FIGURE 4:**
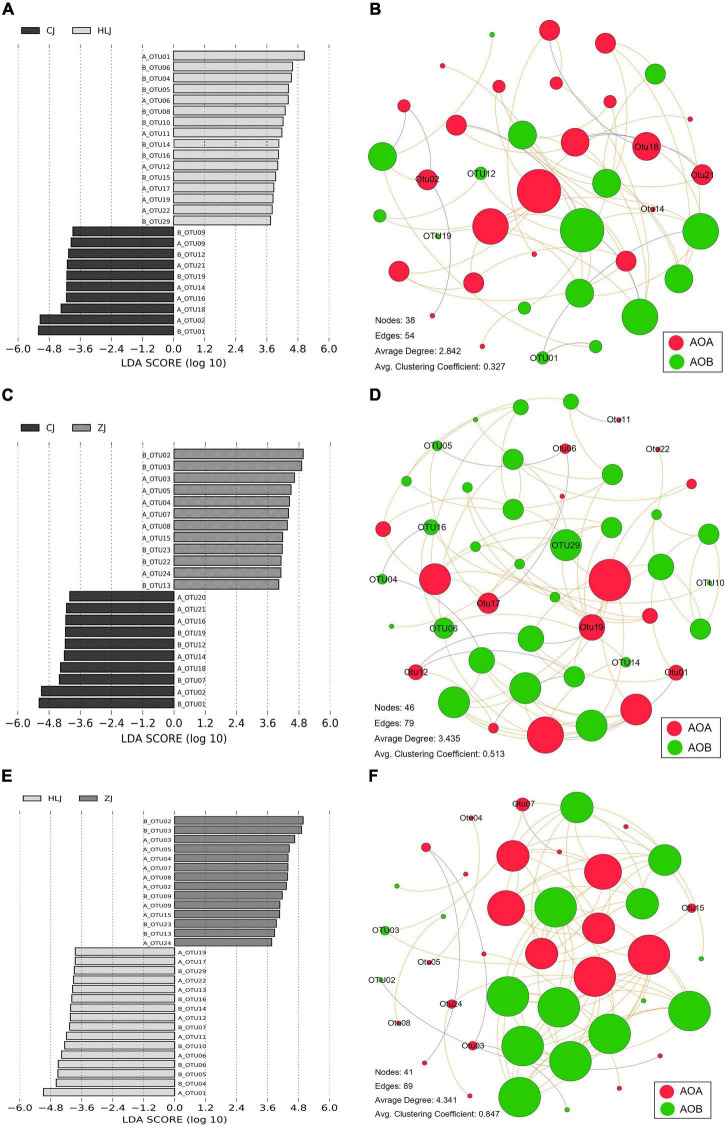
Histogram of linear discriminant analysis scores computed for differentially abundant ammonia-oxidizing archaea (AOA) and ammonia-oxidizing bacteria (AOB) operational taxonomic units (OTUs) **(A,C**,**E)**, and their co-occurrence networks in different sampling areas (**B**,**D**,**F,** representing CJ, HLJ, and ZJ areas, respectively). A_ and B_ represent AOA and AOB OTUs, respectively. OTUs with alpha values for the factorial Kruskal-Wallis test of less than 0.05, and LDA scores > 2.0, are shown. Nodes of different colors indicate different microbial communities, and the sizes are proportional to the connection numbers. Edges indicate correlations between different nodes, with the positive and negative correlations represented in red and green colors, respectively. Only the differentially abundant OTUs in different areas are indicated.

The AOA OTUs could be attributed to four groups, forming six clusters (Figure 3A), and the AOB OTUs could be attributed to two groups, forming seven clusters (Figure 3B). No significant difference was found between AOA and AOB OTUs from different sampling times in CJ, HLJ, or ZJ ponds according to the analysis of LDA Effect Size; however, differentially abundant AOA and AOB OTUs were detected in different sampling areas (Figure 4). Specifically, five OTUs mainly belonging to Cluster 1, seven OTUs mainly belonging to Clusters 1 and 2, and seven OTUs mainly belonging to Cluster 3, in the AOA *Nitrososphaera* Group 1.1b were found to be differentially abundant in CJ, HLJ and ZJ pond sediments, respectively, compared to other areas. Similarly, three OTUs mainly belonging to Cluster 4 and seven OTUs mainly belonging to Cluster 1 in *Nitrosospira*, and four OTUs mainly belonging to Cluster 7 in *Nitrosomonas*, were differentially abundant in CJ, HLJ, and ZJ ponds, respectively, compared to other areas. In addition, co-occurrence network analysis indicated that most of these differentially abundant OTUs tended to be the keystone taxa in structuring AOA and AOB communities in aquaculture pond sediments (Figure 4).

### Relationships between ammonia-oxidizing archaea and ammonia-oxidizing bacteria communities and sediment properties

**FIGURE 5:**
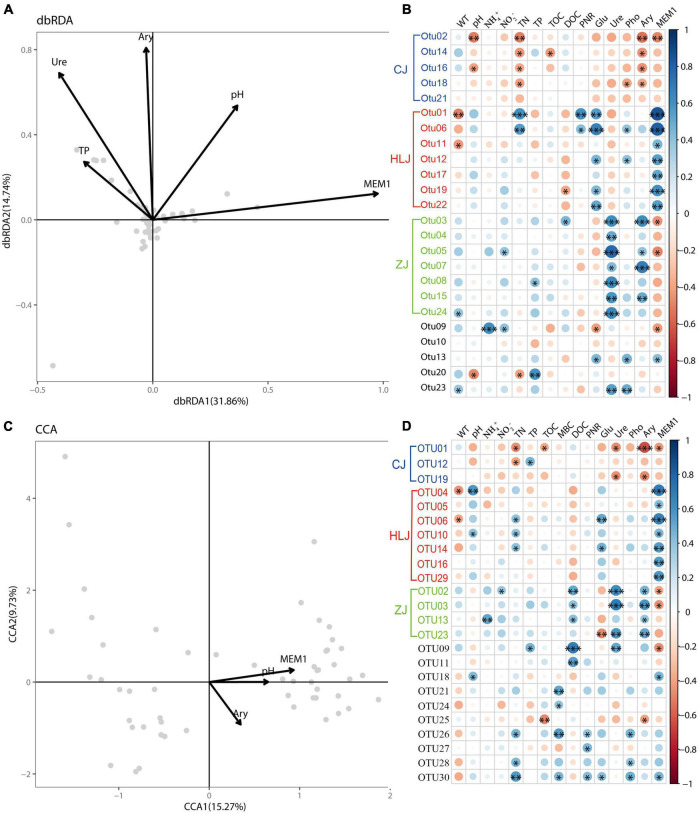
Relationship between sediment properties and the spatial factor MEM1 and ammonia-oxidizing archaea (AOA) and ammonia-oxidizing bacteria (AOB) communities: distance-based redundancy analysis (db-RDA) **(A)** and heatmap **(B)** for AOA communities; canonical correlation analysis (CCA) **(C)** and heatmap **(D)** for AOB communities. Only values with a correlation efficient > 0.6 or *p* value < 0.05 are presented. The spatial factor MEM1 was generated by distance-based Moran’s eigenvector maps. The differentially abundant OTUs between different areas are indicated in different colors: CJ–blue, HLJ–red, and ZJ–green. The asterisks indicate significant correlations, with *P-value < 0.05, ^**^P-value < 0.01, ^***^P-value < 0.01.

Results of db-RDA and CCA showed significant correlations between sediment properties and AOA and AOB communities, respectively, in aquaculture ponds (Figures 5A,C). About 46.60% of AOA variation and 25.00% of AOB variation could be explained by the first two axes of db-RDA and CCA plots, respectively. AOA OTUs were found to be significantly correlated with TP, Ure, Ary, pH, and MEM1, while AOB OTUs were significantly correlated with Ary, pH, and MEM1, indicating significant effects of environmental and spatial factors on ammonia oxidizers in aquaculture pond sediment. Heat maps based on Pearson correlation also indicated significant correlations between OTUs and sediment properties (Figures 5B,D). The most correlated parameters for AOA OTUs were TN, Glu, Ure, Ary, and MEM1, while the most correlated parameters for AOB OTUs were TN, DOC, Ure, Ary, and MEM1. In addition, PNRs were found to be significantly positively correlated with *amoA* gene copy numbers (*p* < 0.05), but negatively correlated with *shannon* and *chao1* (*p* < 0.05) of AOA (Supplementary Figure 3). However, no significant correlation was found between PNRs and AOB.

Interestingly, among the OTUs that were found to be significantly correlated with sediment properties, the differentially abundant OTUs seemed to be prominent and correlated with different environmental factors (Figure 5). Specifically, for AOA the CJ-specific OTUs were mainly influenced by TN and Ary, the HLJ-specific OTUs were mainly influenced by Glu and MEM1, while the ZJ-specific OTUs seemed to be influenced mainly by Ure and Ary. Similar differences were also found for AOB OTUs. For example, the CJ-specific AOB OTUs were mainly correlated with TN, Ure, and Ary, the HLJ-specific AOB OTUs were mainly correlated with pH, TN, Glu, and MEM1, while the ZJ-specific OTUs seemed to be influenced mainly by DOC, Ure, and Ary. However, less correlation was found between sediment properties and other OTUs.

### Process of ammonia-oxidizing archaea and ammonia-oxidizing bacteria community assembly in aquaculture pond sediment

**FIGURE 6:**
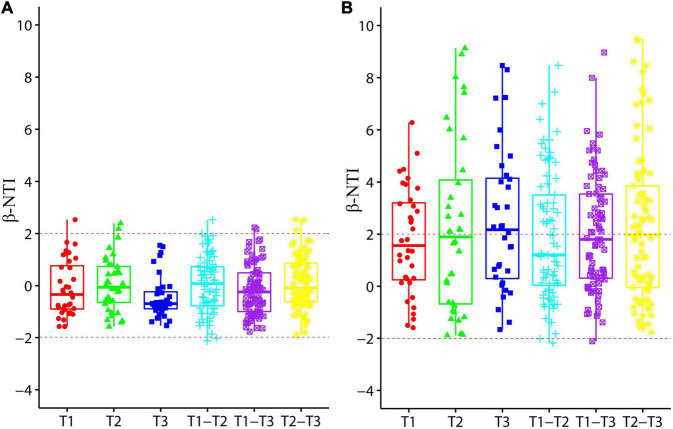
Box plots representing values of β-nearest taxon index (β-NTI) for ammonia-oxidizing archaea (AOA) **(A)** and ammonia-oxidizing bacteria (AOB) **(B)** communities from different sampling times. T1, T2, and T3 represent community comparison at three different sampling times, and T1-T2, T1-T3, and T2-T3 represent community comparison between different sampling times.

Values of β-NTI was calculated to reveal the AOA and AOB community assembly processes in aquaculture pond sediment (Figure 6). Values of AOA communities from different sampling times were well within −2 to 2, and β-NTI values between different sampling times were also concentrated in the range between −2 and 2, indicating an obvious stochastic assembly process in AOA communities. However, β-NTI values of AOB communities were mainly distributed around 2, suggesting that both stochastic and deterministic processes could be important for AOB community assembly in aquaculture pond sediment.

## Discussion

### Dynamics of ammonia-oxidizing archaea and ammonia-oxidizing bacteria communities in aquaculture pond sediment

Ammonia-oxidizing microorganisms have been studied for decades with the help of different sequencing methods. Although several primers have been used to study ammonia oxidizers with the high throughput methods, their reliability and comparison with previous studies in different environment have seldom been researched. In this study, we chose the clone library method to study ammonia oxidizers in aquaculture ponds, because it has been well-established for study of both AOA and AOB with the widely used *amoA* gene primers (Rotthauwe et al., 1997; Francis et al., 2005), and produce much longer fragments. The results could also be easily compared to previous studies of ammonia oxidizers in aquaculture ponds, which have also used the clone library method (Lu et al., 2015; Dai et al., 2018). Despite the massive research, the actual dynamics of AOA and AOB communities in environments especially when subjected to disturbance are still not very clear, probably due to their complex responses to environmental changes at both temporal and spatial scales. Studies of different layers in water bodies have shown significant seasonal changes in AOA and AOB communities (Vissers et al., 2013; Muller et al., 2018), while many other studies have discovered no or little seasonality in ammonia oxidizers in the sediments of estuaries (Zheng et al., 2014; Smith et al., 2015), lakes (Liu and Yang, 2021), natural wetlands (Wang et al., 2013; Gao et al., 2018), constructed wetlands (Wang et al., 2019), and biofilms (Bagchi et al., 2014), indicating that the responses of ammonia-oxidizing communities to seasonal variables may differ. Two previous studies have observed some seasonal trends in abundances of ammonia-oxidizing microorganisms in aquaculture ponds (Kumari et al., 2011; Lu et al., 2015), but no significant seasonal differences could be identified because there were not enough samples for statistical analysis. In this study, using aquaculture ponds in different areas, we identified some significant temporal differences of *amoA* gene abundances and diversity of AOA and AOB in certain ponds that we sampled, which is consistent with previous studies. However, no significant differences were found in other ponds, indicating complex community dynamics of AOA and AOB communities in aquaculture ponds, and that the results from certain ponds could vary. Further, no significant differences of community composition of AOA and AOB were found between different sampling times, suggesting no overall temporal dynamics in the AOA and AOB communities in the aquaculture ponds. Our results emphasized the importance of comprehensive analyses of AOA and AOB communities from different aquaculture ponds when determining their temporal dynamics.

In contrast, significant differences in *amoA* gene abundances and diversity of AOA and AOB communities were found between the sampling areas in our study. Significant differences of AOA and AOB community compositions were also found between sampling areas, indicating spatial variations of ammonia oxidizers in aquaculture ponds. Although AOA and AOB are reported as being globally distributed (Martiny et al., 2011; Cao et al., 2013), significant differences in their regional patterns have been detected. For example, significant regional differences in AOA and AOB community composition have been observed in salt marsh sediments (Martiny et al., 2011; Bernhard et al., 2019), sea water (Sintes et al., 2015), forest soil (Chen et al., 2015), and other environments, indicating apparent effects of regional conditions on ammonia-oxidizing communities. Our results provide further evidence for the effect of spatial variables on AOA/AOB communities. In addition, our results also indicated a higher regional divergence of AOA compared to AOB, according to the PCoA, probably due to their lower dispersal at a regional scale (Sintes et al., 2015). Furthermore, no clear distance-decay relationships, which exist in other microorganisms (Bell, 2010; Zinger et al., 2014), were found for AOA and AOB communities in our study, since no additional differences were found between HLJ and ZJ samples compared to others (the distance between HLJ and ZJ ponds is much greater than distances between HLJ and CJ, and between CJ and ZJ ponds).

Previous studies have detected distinct community compositions of ammonia oxidizers at regional scales (Bernhard et al., 2019; Cardarelli et al., 2020). Consistent with these studies, we found significantly different community compositions of AOA and AOB in different areas, and identified differentially abundant OTUs in aquaculture ponds from different areas (see Figure 4). These area-specific OTUs contained taxa that were dominant and closely connected to other taxa in AOA/AOB communities, which indicate that they might significantly contribute to the regional variations of AOA and AOB communities in aquaculture ponds. On the other hand, the area-specific OTUs were not clearly separated according to phylogeny (see Figure 3) despite their regionally distinct distribution patterns. This result demonstrates that geographic distance did not result in any clear evolutionary divergences in ammonia oxidizers in the aquaculture ponds. Indeed, a number of studies have indicated that the effect of geographic distance on community similarity may not necessarily lead to evolutionary divergent provinces (Heino et al., 2010; Martiny et al., 2011; Wan et al., 2021). Local determinants and intrinsic differences between different microorganisms could contribute to locally distinct communities, and thus determine their local and regional patterns (Sintes et al., 2015; Bernhard et al., 2019).

### Factors influencing ammonia-oxidizing archaea and ammonia-oxidizing bacteria community dynamics in aquaculture pond sediment

Temperature has been considered to be an important factor influencing the seasonal patterns of ammonia oxidizers (Biller et al., 2012; Gao et al., 2018). In this study, however, there seemed to be little influence of temperature on AOA and AOB community dynamics (Figure 5), which probably contributed to the non-significant temporal variation in AOA and AOB communities in aquaculture ponds, especially considering that the water temperature changed significantly between different sampling times. On the other hand, concentrations of TN and TOC significantly changed between sampling times in our study, due to aquaculture activities that produced large amounts of organic deposits in the sediment, and significant relationships were found between TN/TOC and AOA/AOB communities. Nevertheless, the effects of TN/TOC on ammonia oxidizers in our study seemed to operate only at regional scale, because the changes in TN/TOC between sampling times did not lead to temporal changes in AOA/AOB communities. Instead, the significant differences in TN/TOC between different areas could have a significant effect on ammonia oxidizers, considering the significant community differences in ponds of different areas. Besides, and in consistent with our previous study (Dai et al., 2018), contents of β-glucosidase, urease, and arylsulfatase were also found to be correlated with AOA and AOB communities. However, the effects seemed to be at a regional scale, and the differences in contents of β-glucosidase, urease and arylsulfatase at a regional scale in the present study could contribute to the significant differences in AOA and AOB communities between different areas. We deduce that although distribution of ammonia oxidizer could be affected by many environmental factors, their dynamics could be relatively independent at particular environment, probably due to the adaption of their communities.

The disturbances of aquaculture activities on pond sediment are reflected not only in nutrient accumulation, but also in the resulting effects of organic matter decomposition. The large amounts of decomposition produces high levels of ammonia, inducing a highly reduced environment, and could also contribute to the sediment variations. Consistent with our previous study, no significant correlation between ammonia concentration and AOA and AOB was found in this study, probably due to the excessive ammonia in aquaculture pond sediment (Dai et al., 2018). Other environmental factors such as pH and TP were found to significantly affect AOA and AOB communities in aquaculture pond sediment in our study, but their effects were only significant for a few OTUs according to correlation analyses. In addition, these factors were not significantly changed between different ponds, indicating few influences of these environmental factors on AOA and AOB communities in aquaculture pond sediment. On the other hand, and perhaps more importantly, geographic distance was found to be significantly correlated with AOA and AOB community composition in our study, which provides further evidence for spatial dispersal limitation in aquaculture ponds of different areas. This factor also appears vital in explaining variations in AOA and AOB abundances (Li et al., 2022). Thus, geographic distance might be an important factor driving dynamics of ammonia-oxidizing archaeal and bacterial communities. Furthermore, in this study, PNRs were found to be significantly correlated with *amoA* gene copy numbers, *shannon* and *chao1* in AOA communities, but not in AOB communities, indicating the important role of AOA in nitrification in aquaculture ponds. Considering the significant differences in AOA in our aquaculture pond sediments, it is possible that nitrification in some aquaculture ponds is inhibited.

Environmental filtering and adaption of the microbial community have been found in many previous studies (Fierer et al., 2009; Pierre et al., 2017). The existence of area-specific taxa of ammonia oxidizers in our study probably indicates filtering or adaption of AOA and AOB communities in the local aquaculture environment. Indeed, environmental factors were mainly correlated with the area-specific taxa, and different responses of these taxa to environmental factors in different sampling areas were also detected (see Figure 5). Thus, if their potentially important contribution to regional community variation is also considered (see discussion above), locally distinct taxa might be important in determining the response of AOA and AOB communities to environmental factors in aquaculture ponds. On the other hand, nearly 53.4% of AOA variation and 75% of AOB variation could not be explained by the environmental factors in our ponds (see Figure 5). One reason could be that we missed an unmeasured variable that contributed to the regional distance effect. In our study, we tried to reduce the environmental variation between different areas by choosing similar aquaculture ponds where the same fish were reared. A second reason could relate to microbial interactions, which have been indicated to be important in structuring microbial communities (Guggenheim et al., 2020). Although the differentially abundant OTUs between different areas tended to be the keystone taxa in structuring AOA and AOB communities in our study (Figure 4), there were also large amounts of other closely related taxa which were common in different areas. These taxa might affect AOA and AOB communities differentially in different areas, and thus might also contribute to unexplained variation. In addition, other microorganisms could also contribute to the unexplained variation. Although MBC was found to have slight effects on ammonia oxidizers in our aquaculture pond sediment, which might be overridden by the effects of other parameters such as Ary, Ure, MEM1, and pH, the particular microbial community present could significantly contribute to the dynamics of oxidizing communities in aquaculture ponds. Further studies on the effect of other microbes on ammonia oxidizers, based on more detailed analyses, are therefore still needed. Last but not least, although we tried our best to make sure that the sequencing depth was sufficient to cover all or nearly all the OTUs in the samples, the limitation of the clone library method could still cause incomplete analyses of the community composition, and thus affect the results of community differences between different samples. More extensive sequencing analyses are thus needed to confirm the results.

### Community assembly processes of ammonia-oxidizing archaea and ammonia-oxidizing bacteria communities in aquaculture pond sediment

In this study, values of β-NTI were evaluated to reveal the AOA and AOB community assembly processes in aquaculture pond sediment. Surprisingly, a clear stochastic process was detected in AOA communities, and a stochastic process was also important in AOB community assembly. Dispersal limitation has been thought to be a vital mechanism of stochastic processes (Liu et al., 2020; Li et al., 2021). Indeed, dispersal limitation, and niche selection have been found to greatly determine the composition of ammonia oxidizers (Martiny et al., 2011; Wang et al., 2019; Li et al., 2022). Significant correlations were observed in our study between geographic distance (MEM1) and AOA/AOB communities, indicating obvious dispersal limitation in aquaculture ponds of different areas. Interestingly, the effect of geographic distance on the community similarity of ammonia oxidizers has been reported to happen at a certain scale (e.g., < 3,000 km), but not at larger scales (Martiny et al., 2011; Sintes et al., 2015). The results of our study revealed significant correlation between geographic distances less than 3,000 km and AOA and AOB communities, and could provide further evidence to support this distribution pattern.

The structural stability of a community indicates its capacity to resist changes when exposed to small biotic or abiotic perturbations (Rohr et al., 2014; Barbier et al., 2018). Instability of community structure has been found to be prevalent in nature, and is often attributed to the impact of environmental change. However, recent studies based on theoretical models and empirical observation have shown that species richness could be intrinsically regulated (for instance by species interaction, and dispersal) at both local and regional scales (O’Sullivan et al., 2019, 2021). In this study, although sediment properties in aquaculture ponds varied significantly between different sampling times, few significant differences were found in AOA and AOB community structures between different times, indicating no or little response of the AOA and AOB community to sediment change at a local scale. Nevertheless, significant differences were found in AOA and AOB communities between different areas, probably suggesting a regional variation in AOA and AOB communities due to dispersal limitation. The area-specific OTUs and their contribution in determining the response of AOA and AOB communities to environmental factors also indicated the significant effects of community composition on AOA/AOB dynamics. In addition, geographic distance was found to be significantly correlated with AOA and AOB community composition, which demonstrates that dispersal limitation could significantly contribute to the variations in AOA and AOB communities, and stochastic processes based on community characteristics were found to be important in structuring AOA/AOB communities in aquaculture ponds. Taken together, our results revealed for the first time the dynamics of ammonia-oxidizing archaea and bacteria based on community characteristics in aquaculture pond sediment. However, further studies of ammonia-oxidizer community dynamics based on more quantitative analyses at different distance scales are still needed to better illustrate their community assembly patterns.

## Conclusion

In this study, we analyzed the spatial and temporal dynamics of AOA and AOB communities based on three different areas, and for the first time determined their community assembly patterns in aquaculture pond sediment. We found some significant temporal changes in AOA and AOB community diversity and abundances, but no temporal changes in community composition, despite the significant variations in sediment properties between different sampling times. The AOA and AOB communities changed significantly between different sampling areas, with distinct area-specific OTUs detected. Sediment properties seemed to affect AOA and AOB communities at a regional scale, indicating a significant role of community composition in AOA/AOB dynamics. Geographic distance was found to be significantly correlated with AOA and AOB community composition, which demonstrates that dispersal limitation could significantly contribute to the variation in AOA and AOB communities. In addition, analyses suggested that stochastic processes play an important role in structuring AOA/AOB communities in aquaculture ponds. We concluded that community characteristics might be important in the dynamics of AOA and AOB in aquaculture pond sediment. Our results, for the first time, provide evidence for the dynamics of AOA and AOB communities being driven by stochastic factors in a disturbed environment, and might also be of use in the management of the aquaculture environment.

## Data availability statement

The datasets presented in this study can be found in online repositories. The names of the repository/repositories and accession number(s) can be found below: https://www.ncbi.nlm.nih.gov/genbank/, ON511010-ON511087; https://www.ncbi.nlm.nih.gov/genbank/, ON510946-ON511009.

## Author contributions

LD designed the study, conducted the experiments, analyzed the data, and wrote the manuscript. LY contributed to data interpretation and revision of the manuscript. LP collected samples and conducted the experiments. YL contributed to the design of the study and revised the manuscript. LT and GL supervised the design and execution of the project. All authors contributed to the manuscript and approved the submitted version.

## Footnotes

1
http://www.ncbi.nlm.nih.gov

2
http://huttenhower.sph.harvard.edu/galaxy
