# Supplementary material for: Stochastic factors drive dynamics of ammonia-oxidizing archaeal and bacterial communities in aquaculture pond sediment
Source: Front Microbiol. 2022 Oct 6;13:950677. doi: 10.3389/fmicb.2022.950677 (PMC9583541; doi:10.3389/fmicb.2022.950677)
Supplement: Supplementary file 1 [file Data_Sheet_1.DOCX]

***Frontiers in Microbiology***

***Supplementary Material***

**Supplementary Figures**

(A) (B)


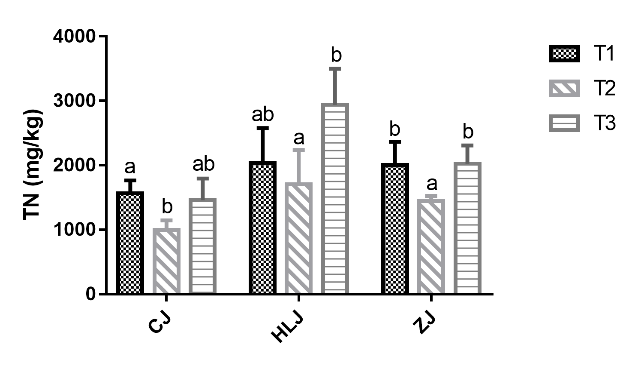

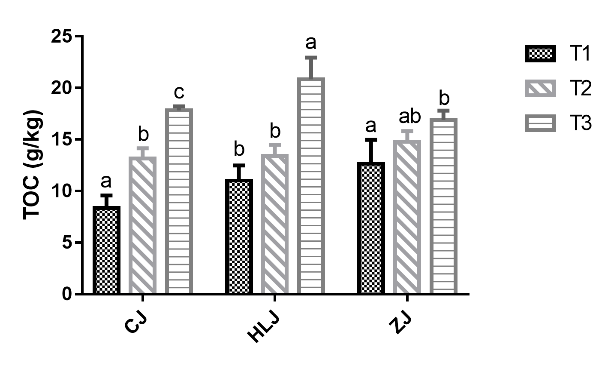


**Figure 1 |** TN **(A)** and TOC **(B)** concentration of aquaculture pond sediment samples at different sampling times. CJ - Changjiang ponds. HLJ - Heilongjiang ponds. ZJ - Zhujiang ponds (different letters above the bars of the same area indicate significant difference at 5% level). T1-3 indicate different sampling times

(A) (B)


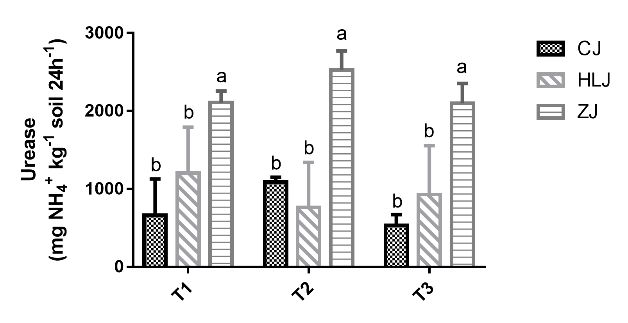

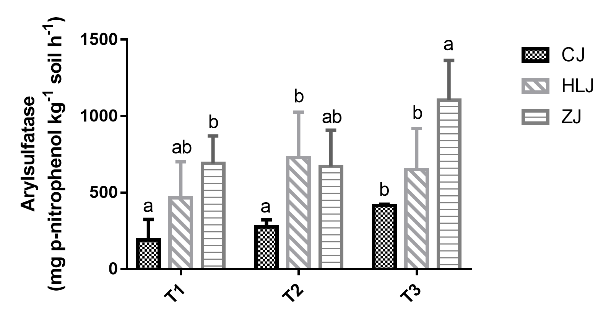


**Figure 2 |** Urease **(A)** and arylsulfatase **(B)** concentration of aquaculture pond sediment samples in different sampling areas. CJ - Changjiang ponds. HLJ - Heilongjiang ponds. ZJ - Zhujiang ponds (different letters above the bars of the same time indicate significant difference at 5% level). T1-3 indicate different sampling times

(A) (B) (C)


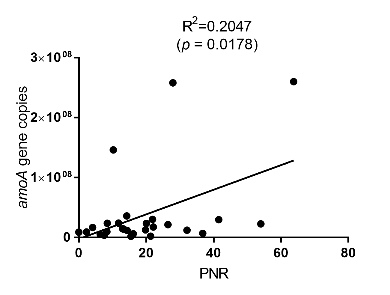

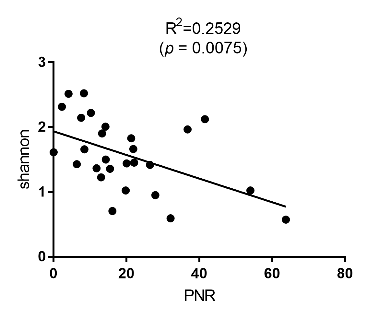

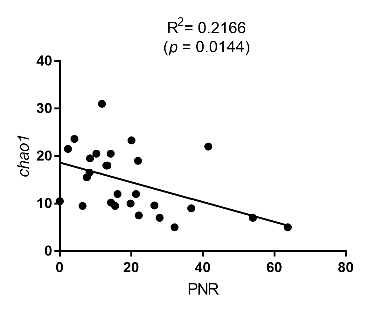


**Figure 3 |** Scatter plot of correlation between potential ammonia oxidation rates (PNRs) and *amoA* gene copies (A), *shannon* (B) and *chao1* (C) of AOA in aquaculture pond sediment
